# Enhancing therapeutic effects of murine cancer vaccine by reshaping gut microbiota with *Lactobacillus rhamnosus* GG and jujube powder

**DOI:** 10.3389/fimmu.2023.1195075

**Published:** 2023-06-26

**Authors:** Nan Jing, Luoyang Wang, Huiren Zhuang, Guoqiang Jiang, Zheng Liu

**Affiliations:** ^1^ Department of Chemical Engineering, Tsinghua University, Beijing, China; ^2^ Key Lab of Industrial Biocatalysis, Ministry of Education, Tsinghua University, Beijing, China; ^3^ School of Basic Medicine, Qingdao University, Qingdao, China

**Keywords:** cancer vaccines, immunotherapy, gut microbiota, synbiotic, nutritional intervention

## Abstract

Cancer vaccines have gained widespread attention in recent years as an emerging treatment for tumors. However, most therapeutic cancer vaccines have failed in phase III clinical trials due to faint clinical benefits. In this study, we funded that a specific synbiotic composing *Lactobacillus rhamnosus* GG (LGG) and jujube powder significantly enhanced the therapeutic effects of whole cells cancer vaccine in MC38 cancer cells bearing-mouse. The utilization of LGG increased the abundance of *Muribaculaceae*, which is conductive to an enhanced anti-tumor effect, but reduced microbial α-diversity. The use of jujube nursed probiotic microorganisms in *Lachnospiaceae* and enriched microbial diversity, as indicated by increased Shannon and Chao index. The reshaped gut microbiota by this synbiotic improved lipid metabolism conductive to intensified infiltration of CD8^+^ T cells in the tumor microenvironment and enhanced the potency of above-mentioned cancer vaccine. These encouraging findings are helpful for further efforts towards enhancing the therapeutic effects of cancer vaccines through nutritional intervention.

## 1 Introduction

Cancer immunotherapy has achieved tremendous advances in many types of cancer in recent years. The therapeutic cancer vaccines, one of the most advanced parts of immunotherapy, have undergone a resurgence in the past decade (1). While vaccines have widely been used to prevent infectious diseases, their ability to elicit and amplify antigen-specific immune responses has also been noted as a potential way to cancer treatment (2, 3). Early therapeutic cancer vaccines focused on antigens overexpressed on cancer cells, which are referred to tumor associated antigens (TAAs). However, this strategy was so far largely disappointing in generating anti-tumor immune responses in clinical trials, and the experimental results are discouraging in terms of a poor responsive rate below 7% and a low overall rate around 20% (4, 5).The discovery of tumor neoantigens resulting from tumor-associated gene mutations has sparked new possibilities for cancer vaccines, propelling the development of personalized cancer vaccines. Short peptides or RNAs targeting tumor neoantigens have proven to be more effective in mouse models (6, 7). A clinical study by Ugur and colleagues demonstrated that mRNA cancer vaccines not only effectively prevented recurrence but also significantly enhanced the anticancer capacity of the patients (8). A preliminary trial of personalized cancer vaccine in melanoma patients conducted by Wu and co-workers also produced encouraging results (9).

On the other hand, growing evidence are reported on the effects of gut microbiota on cancer immunotherapy using immune checkpoint blockade (ICB) (10–12) and adoptive immune cell transfer (ATC) (13, 14). Specific microorganisms that are associated with the improved anticancer effects of ICB has been identified (10–12). As to ATC, Paulos and co-workers have revealed that microbiota-depleted mice had impaired anticancer efficacy due to decreased levels of activated dendritic cells and functionally impaired CD8^+^ T cells (13). The specific gut bacteria *Bacteroidetes*, including *Bacteroides* and *Parabacteroides*, have been found to enhance CD8^+^ dendritic cells, which sustained the adoptively transferred anti-tumor T cells in an IL-12-dependent manner (14). However, the effect of gut microbiota on cancer vaccines still remains elusive.

It is established that foods and dietary habits impact the abundance of gut microorganisms and thus the overall health (15). Recent years have witnessed growing efforts in developing medical function foods for patient care and treatment (16), in which a combination of probiotic and prebiotic (also termed as synbiotic) is of particular note for its high capability in reshaping gut microbiota. Nam and colleagues showed the inhibiting effect of a synbiotic containing *Lactobacillus gasseri* 505 and *Cudrania tricuspidata* leaf extracts against the colitis-associated colorectal cancer (17). Besides, Kacy and co-workers found that a synbiotic of *Lactobacillus rhamnosus* GG (LGG) and dietary fiber regimen inhibited the growth of colorectal cancer by downregulating the genes involved in procardiogenic pathways, drug resistance and the levels of the oncometabolite lactate (18).

*Ziziphus jujuba* Mill (syn. jujube or Chinese date), has been cultivated and consumed in China for over 4,000 years and is a popular fruit in many other Asian countries as well. Jujube has been widely reported to have plentiful health benefits resulting from their polyphenols, flavonoids and other bioactive substances. Recent research has also suggested that jujube has potential prebiotic effects on gut microbiota because of its high content of carbohydrates (19). Studies have shown that oral supplement with jujube powder increased the abundance of beneficial bacteria in gut, such as *Lachnospiraceae*, *Ruminococcaceae* and *Bifidobacteriales*, and elevated the production of short chain fatty acids, which improved the effectiveness of cancer immunotherapy and chemotherapy (20, 21). Our previous studies have demonstrated that full-component jujube powder appeared superior performance, as compared to its specific components, in inhibiting tumor growth combining with anti-programmed death-ligand 1 (PD-L1) blockade treatment (20, 22). Hence, we speculate that jujube powder holds great potential as prebiotics. *Lactobacillus rhamnosus* GG (LGG) has been shown in numerous studies to be beneficial to the anti-tumor immune response. Xing and colleagues reported that oral administration of LGG reduced the abundance of *Verrucomicrobia* while increased the abundance of *Bacteroidetes* containing *Lactobacillus rhamnosus* GG and *Bacteroides uniformis*, both of which are favorable for the anti-Programmed cell death 1 (PD-1) immunotherapy by reinforcing dendritic cells and CD8^+^ T cell infiltration in tumors (23). Owens et al. found that oral treatment with LGG reduced tumor load in Azoxymethane (AOM)/Dextran Sodium Sulfate (DSS)-induced colon cancer mice in a CD8^+^T cell-dependent manner (24).

Here, we propose a new synbiotic composed by *Lactobacillus rhamnosus* GG (LGG) and jujube, termed as LGG-JP hereafter, and test its capability of reshaping gut microbiota for cancer treatment with whole cells cancer vaccine.

Whole cells cancer vaccine represents one form of cancer immunotherapy currently in development and clinical trials. A significant advantage of this cancer vaccine is that the tumor cells provide a broad range of potential antigens, eliminating the need to identify a single optimal antigen for targeting a specific type of cancer. Besides, this approach can generate immune responses to multiple tumor antigens, thus bypassing issues of tumor antigen loss. In this work, we investigated the effect of reshaping gut microbiota with LGG and jujube powder on the therapeutic effects of whole cells cancer vaccine produced by mice MC38 cells on mice colon cancer. These findings have the potential to inform future research into other types of cancer vaccines. The lymphocyte infiltration in the tumor microenvironment of mice was analyzed to evaluate the anti-tumor immune response. The 16S rRNA gene sequencing and untargeted metabolomics analysis of mice feces were performed to profile the effect of gut microbiota on anti-tumor immune response of cancer vaccine. The results obtained in this work suggested the potential of manipulating gut microbiota through natural nutrients and probiotics for patients treated with cancer vaccine.

## 2 Materials and methods

### 2.1 Materials

Jujube powder was prepared in our laboratory according to previous studies (20), in which jujube powder with the particle size under 10 μm was used in the present study. The chemical composition and physical characteristics of jujube powder can be found in our previous studies (20, 22). *Lactobacillus rhamnosus* GG (LGG) lyophilized powder (Total living bacteria count>1.0×10^10^ CFU/g) was purchased from ZHONGKE-JIAYI (Shandong Zhongke Jiayi Biological Engineering Co., Ltd, Weifang, China). *In vivo* anti-mouse PD-L1 (BE0101) mAb and immunoglobulin G2b isotype (IgG2b, BE0090) were purchased from BioXCell (West Lebanon, NH, USA). Mouse antibodies for flow cytometry were obtained from BioLegend (San Diego, CA, USA). Dulbecco’s modified eagle medium (DMEM) culture medium, Fetal Bovine Serum (FBS), Penicillin-Streptomycin (10,000 U/mL) solution and Phosphate Buffer Saline (PBS) were purchased from Gibco (Grand Island, NY, USA). All antibiotics were purchased from Invitrogen (Carlsbad, CA, USA).

### 2.2 Preparation of whole cells cancer vaccine

Mouse colon cancer MC38 cells were procured from the American Type Culture Collection and cultivated in DMEM culture medium containing 10% FBS and 1% Penicillin-Streptomycin solution. The cells were maintained at 37 °C with a 5% CO_2_ atmosphere. Inactivated cell lysates were prepared by high-salt dehydration, liquid nitrogen freeze-thawing, ultrasonication and X-ray irradiation to produce whole tumor cell vaccines. In detail, MC38 cells in logarithmic growth phase were digested with 0.25% Trypsin-EDTA (1×) (Gibco, Grand Island, NY, USA) for 5 min and centrifuged to obtain cell precipitates (450 g, 5 min). For the high-salt dehydration method, the cells were directly suspended with PBS (10×) and the cell density was adjusted to 1.0×10^7^ cells/mL. For the other methods, the cell precipitates were resuspended with PBS (1×) and adjusted to 1.0×10^7^ cells/mL, followed by different cell inactivation methods. For X-ray irradiation method, an X-ray irradiator (RS 2000 Pro, RadSource, Brentwood, USA) was used at the intensity of 35 Gy. An ultrasonic cell crusher (JY 92-II, Scientz Biotechnology Co., Ltd, Ningbo, China) was employed for ultrasonication method with 3s of working time and 3s of interval for 150 times. In liquid nitrogen freeze-thawing method, the cell suspension was put into the liquid nitrogen tank for 2 min, followed by 37 °C water bath for 1 min immediately. This operation was repeated for 5 times. The prepared cancer vaccines were stored at -80 °C.

### 2.3 Animal studies

Female C57BL/6 mice (7-week old) were purchased from Vital River (Beijing, China) and kept in specific pathogen free conditions in accordance with protocols approved by Institutional Animal Care and Use Committees of Tsinghua University (Approval ID 20-LZ1-1).

Pre-treatment animal experiment: Following one week of adaptation, mice were allocated into various groups using randomization (n=6). Different abbreviations are used here to represent vaccines prepared by diverse approaches. Mice in experimental groups were subcutaneously immunized three times at the left flank on day 0, 7, and 14 with 100 μL of whole cells cancer vaccines produced by high-salt dehydration (Group abbreviation: PB), liquid nitrogen freeze-thawing (Group abbreviation: LN group), ultrasonication (Group abbreviation: US) and X-ray irradiation (Group abbreviation: XR), respectively, while the control group (CTR) received sterile PBS (1×) at the same time. On day 21, the density of MC38 cells at the logarithmic growth phase was adjusted to 5×10^6^ cells/mL. Then, 100 μL of the cells were subcutaneously inoculated at the right flank of all mice. Tumor volumes were determined twice a week using the formula: (length × width^2^)/2. Mice were euthanized when the tumor volume exceeded 2000 mm^3^ or any side length exceeded 20 mm as a humanitarian endpoint to plot survival curves.

Pre-treatment animal experiment with antibiotic cocktail: Following one week of adaptation, mice were allocated into various groups using randomization (n=6). Mice in experimental groups were subcutaneously immunized at the left flank on day 0, 7, and 14 with 100 μL of whole cells cancer vaccines produced by X-ray irradiation (Group abbreviation: Pre-treated Vaccine). The group treated with an antibiotic cocktail (Abx) had ampicillin (1 g/L), neomycin (1 g/L), metronidazole (1 g/L), and vancomycin (0.5 g/L) added to their drinking water from day 0 to day 49 (Group abbreviation: Pre-treated Vaccine+Abx). Similarly, these mice (Pre-treated Vaccine+Abx) were also subcutaneously immunized at the left flank on day 0, 7, and 14 with 100 μL of whole cells cancer vaccines produced by X-ray irradiation. On day 21, the density of MC38 cells at the logarithmic growth phase was adjusted to 5×10^6^ cells/mL. Then, 100 μL of the cells were subcutaneously inoculated at the right flank of all mice.

Post-treatment animal experiment: On day 0, the density of MC38 cells at the logarithmic growth phase was adjusted to 5×10^6^ cells/mL. Then, 100 μL of the cells were subcutaneously inoculated into the right flank of all mice and the mice were allocated into various groups using randomization (n=6) based on tumor size on day 7. Mice in experimental groups (Post-treated Vaccine) received subcutaneous immunizations on day 7, 14, and 21 with 100 μL of whole cell cancer vaccines produced by X-ray irradiation at the left flank. The control group (CTR) received sterile PBS (1×) at the same time.

Animal experiments with synbiotic treatment: On day 0, the density of MC38 cells at the logarithmic growth phase was adjusted to 5×10^6^ cells/mL. Then, 100 μL of the cells were subcutaneously inoculated into the right flank of all mice and the mice were allocated into various groups using randomization (n=6) based on tumor size on day 7. Mice in experimental groups (including group V, VJP, VLGG and VLGG-JP) received subcutaneous immunizations on day 7, 14, and 21 with 100 μL of whole cells cancer vaccines produced by X-ray irradiation at the left flank. The control group (CTR) received sterile PBS (1×) at the same time. Mice in the jujube powder-treated group were given 800 mg/kg of jujube powder *via* oral gavage every day, starting from day 7 until day 28 (Group abbreviation: VJP). For the group treated with *Lactobacillus rhamnosus* GG (LGG) (Group abbreviation: VLGG), mice were oral gavaged with 100 μL of LGG suspension (2.0×10^9^ CFU/mL) from day 7 to day 28. For the group treated with the symbiotic (LGG-JP) composing jujube powder and LGG (Group abbreviation: VLGG-JP), mice were gavaged with 100 μL of mixture from day 7 to day 28, with the final dose being the same as when used alone. All reagents were prepared daily in distilled deionized water.

### 2.4 Analysis of tumor infiltrating lymphocytes

The infiltration of lymphocytes in tumor tissue was analyzed by flow cytometry, as described elsewhere (20). To generate single cell from the tumor tissue (100-200 mg), Collagenase IV (Yeasen Biotech, Shanghai, China) was employed to digest the tissue in HBSS at 37°C for 30 minutes. The single cells were obtained by filtering the mixture through a 70 μm cell strainer (BD, Franklin Lakes, USA). Then, surface staining with anti-mouse antibodies for CD45 (GK1.5), CD4 (30-F11) and CD8 (53-6.7) was carried out on the single cells in the dark at 4°C for 30 min. After washing twice with PBS, the stained cells were fixed in a Foxp3/Transcription Factor Staining Buffer Set (eBioscience, San Diego, USA) in the dark at 4°C for 30 min. Subsequently, the fixed cells were permeabilized and stained with antibodies for IFN γ (XMG1.2) in the dark at 4°C for 30 min. Finally, the cells were washed twice, suspended in fluorescence-activated cell sorting (FACS) buffer, and analyzed using a FACS Calibur flow cytometer (BD, Franklin Lakes, USA). FlowJo software (TreeStar, Ashland, USA) was utilized to interpret the results.

### 2.5 Untargeted metabolomics analysis of faeces

The untargeted metabolomics analysis of mice feces was conducted by Majorbio Bio-Pharm Technology Co., Ltd. (Shanghai, China) based on standard protocol. Firstly, 50 mg faecal sample was accurately weighted and transferred to a 2 mL centrifuge tube containing grinding beads and 400 μL of extract solution (Methanol: H_2_O = 4:1, v/v). The tube was then frozen and ground for 6 min at -10°C with a frequency of 50 Hz. After low-temperature ultrasound treatment for 30 min (5°C, 40 KHz) and allowing it to stand for 30 min at -20°C, the samples were centrifuged for 15 min (13000 g, 4°C), and the supernatant was taken for analysis. Next, the analysis was performed using HPLC-MS/MS (UHPLC-Q Exactive, Thermo Fisher Scientific, Waltham, USA). Detailed parameters of chromatograph can be found in the literature (25). The elution procedure of mobile phase and the mass spectrometry are listed in **Supplementary Materials** (**Table S1**, **S2**). The original data were analyzed with the Progenesis QI (Waters Corporation, Milford, USA), and metabolic information database (http://www.hmdb.ca/, https://metlin.scripps.edu/) was used to identify metabolite. The mass spectrum quality error is set to less than 10 ppm.

### 2.6 16S rRNA gene sequencing of mice fecal microbiota

On day 28 of the therapeutic cancer vaccines experiment, fecal samples were collected from the mice and genomic DNA was extracted using the QIAamp DNA Stool Mini Kit (Qiagen, Germany) following the manufacturer’s instructions. The gut microbiota composition in the fecal samples was determined through 16S rRNA gene amplicon sequencing. The V3-V4 hypervariable region of the 16S rRNA gene was amplified using universal primers: 338F (5’-ACTCCTACGGGAGGCAGCA-3’) and 806R (5’-GGACTACHVGGGTWTCTAAT-3’) through PCR (ABI GeneAmp 9700). The PCR reaction system (20 μL) consisted of 4 μL 5×FastPfu Buffer, 2 μL 2.5mM dNTPs, 0.8 μL Forward Primer (5 μM), 0.8 μL Reverse Primer (5 μM), 0.4 μL FastPfu Polymerase, 0.2 μL BSA, and 10 ng template DNA (TransGen, China). The amplification procedure included an initial denaturation at 95°C for 3 minutes, followed by 27 cycles of denaturation at 95°C for 30 seconds, annealing at 55°C for 30 seconds, elongation at 72°C for 45 seconds, and a final extension at 72°C for 10 minutes. The PCR products were analyzed using 2% agarose gel electrophoresis, purified with the AxyPrep DNA Gel Extraction Kit (Axygen Biosciences, USA), and quantified with QuantiFluor-ST (Promega, Madison, USA). Subsequently, the products were sequenced using an Illumina MiSeq platform following standard protocols provided by Shanghai Majorbio Bio-Pharm Technology Co. Ltd (Shanghai, China). High-quality sequences were clustered into operational taxonomic units (OTUs) based on 97% similarity. The taxonomic classification of each OTU representative sequence was analyzed using the RDP Classifier against the Silva Database (Release 128, www.arb-silva.de) with a confidence threshold of 0.7. Alpha diversity, represented by the shannon index, simpson index, ace index, and chao index, was calculated using the vegan package in R software. Principal co-ordinates analysis (PCoA) based on Bray-Curtis distance algorithm was performed using the vegan package in R software (version 3.3.1). Circos analysis of samples and species was conducted using Circos-0.67-7 (http://circos.ca/). Permutational multivariate analysis of variance of Bray-Curtis distances was performed using the vegan package. Statistical analysis of taxonomic and functional profiles (STAMP) was performed using the STAMP v2.1.3 software. The functional profiling of gut microbiota was predicted using the R package PICRUSt.

### 2.7 Statistical analysis

The statistical analysis was conducted using GraphPad Prism 8.0 (GraphPad Software Inc., San Diego, USA). Differences between two groups were assessed using the two-tailed Student’s *t*-test if the data is homogenous and followed a normal distribution. If the data is not normal (Kolmogorov-Smirnov test) or not homogenous, samples were analyzed with Mann–Whitney’s U test. The mean and standard error of the mean (SEM) were used to present all graphs. The threshold for statistical significance was set at *p* < 0.05 (* *p* < 0.05, ** *p* < 0.01, *** *p* < 0.001, ns: not significant).

## 3 Results

### 3.1 Effects of gut microbiota on the therapeutic effects of whole cells cancer vaccine treatment

**Figure 1:**
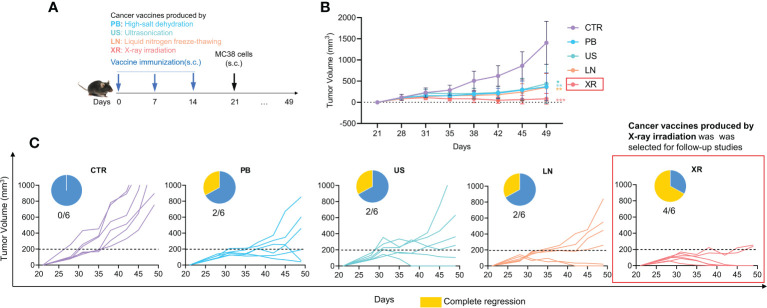
The anti-tumor effects of pre-treated cancer vaccines prepared by different inactivated methods. **(A)** Animal experiment design; **(B)** The tumor growth curve; **(C)** Individual tumor growth curve and complete regression in each group.

The whole cells cancer vaccine contains a broad range of tumor-associated antigens (TAA), and is enriched with antigenic epitopes of CD8^+^ T cells CD4^+^ T cells and thus elicits a comprehensive and effective anti-tumor response (26). Studies have shown that the preventive effect of cancer vaccine is superior to the therapeutic effect, so we examined the effectiveness of inactivation methods on a Pre-treatment model firstly (**Figure 1A**). The experimental groups were named as: Control group (CTR), High-salt dehydration (PS group), Liquid nitrogen freeze-thawing (LN group), Ultrasonication (US group) and X-ray irradiation (XR group). As can be seen from **Figure 1B**, the tumor growth rates are significantly inhibited in all vaccine groups compared with control group, and the tumor volumes are reduced by 73.85% (PB group), 68.86% (US group), 74.78% (LN group) and 94.16% (XR group) respectively compared with CTR group on day 49. Among them, the XR group exhibits the most remarkable effect. **Figure 1C** shows the individual tumor growth curve. One-third (2/6) of the mice in the PB, US and LN groups receive complete regression. Of note, the complete regression in the XR group is two-thirds (4/6). These results indicate that the cancer vaccine prepared by X-ray irradiation shows the best anti-tumor effect. Therefore, the cancer vaccines used in subsequent all studies were all obtained by X-ray irradiation, described as ‘Vaccine’.

**Figure 2:**
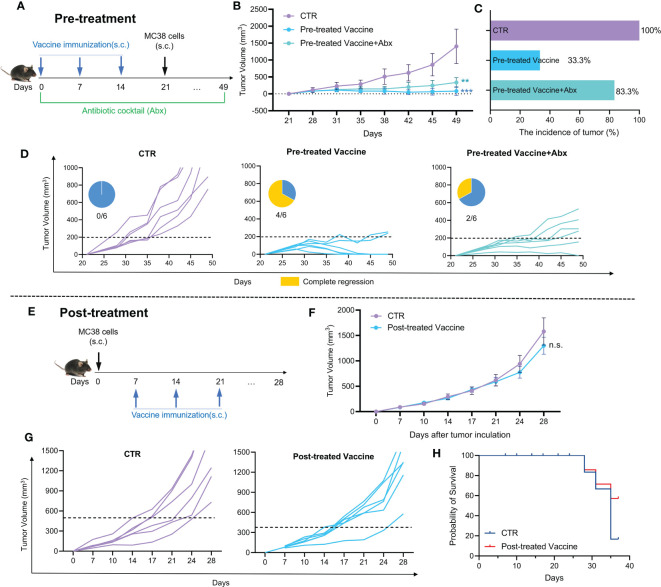
The efficacy of cancer vaccine and their susceptibility to modulation by gut microbiota. **(A)** Pre-treatment animal experiment design; **(B)** The tumor growth curve of pre-treatment animal experiment; **(C)** The incidence of tumor; **(D)** Individual tumor growth curve and complete regression in each group (Pre-treatment). **(E)** Post-treatment animal experiment design; **(F)** The tumor growth curve of post-treatment animal experiment; **(G)** Individual tumor growth curve in each group (Post-treatment); **(H)** Probability of survival (Post-treatment).

Then, the possible role of gut microbiota in Pre-treatment with cancer vaccine was investigated. The animal experiment design is shown in **Figure 2A**. Mice in the experimental group (Pre-treated Vaccine group) were immunized with cells vaccine obtained by X-ray irradiation on day 0, 7 and 14, respectively. For those treated with antibiotic cocktail (Abx), mice received drinking water including different antibiotics throughout the experiment (Pre-treated Vaccine+Abx group). After three times of immunization, all mice were subcutaneously inoculated with MC38 cells on day 21.

It is shown by **Figure 2B** that the inhibition effect is weakened in Pre-treated Vaccine+Abx group, as compared with the Pre-treated Vaccine group. The tumor incidence rate in the Pre-treated Vaccine group was only 33.3%, while that in the Pre-treated Vaccine+Abx group was 83.3% (**Figure 2C**). Moreover, the Abx treatment noticeably reduces the complete remission rate by only 2/6 compared to 4/6 in the Pre-treated Vaccine group (**Figure 2D**). The findings presented in **Figure 2** indicate the indispensable role of gut microbiota in cancer vaccine treatment. It is evident that the depletion of gut microbiota not only hampered the complete regression of the cancer vaccine but also increased the incidence of tumor.

Although pre-treatment with cancer vaccines has shown strong inhibition of tumor growth, the more important role of cancer vaccines is their therapeutic effect on solid tumors. Thus, the therapeutic effect of cancer vaccine was examined (**Figure 2E-H**). The experimental design depicted in **Figure 2E** involved the random division of mice into different groups of six based on their tumor size one week after MC38 cell inoculation. Meanwhile, the experimental mice were immunized with whole cells cancer vaccine for three times respectively. There is no notable disparity in tumor volume between the CTR and Post-treated Vaccine group, though the final tumor growth rate in Post-treated Vaccine group became lower than that in CTR group (**Figure 2F**). Both groups have a similar complete remission with no obvious difference (**Figure 2G**). As shown in **Figure 2H**, the percentage of survival rate in the Post-treated Vaccine group is higher than in the CTR group, indicating the potential of cancer vaccines.

### 3.2 The synbiotic LGG-JP enhanced the therapeutic effects and the infiltration of lymphocytes in the tumor microenvironment

Oral supplementation with probiotics or prebiotics is an effective strategy to improve the intestinal microecology, and we conjecture that such a synbiotic might be an approach to cancer treatment with vaccine. Thus, we examined the therapeutic effects of synbiotic LGG-JP with cancer vaccine.

**Figure 3:**
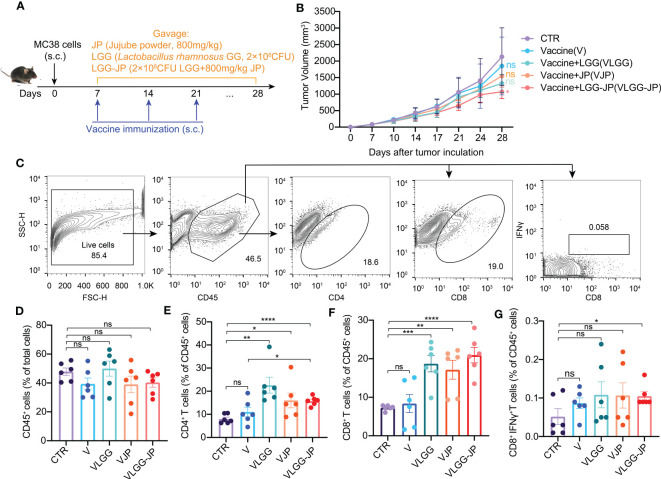
The synbiotic LGG-JP enhanced the response efficiency of cancer vaccines. **(A)** Experimental design of oral supplement jujube powder and/or LGG in combination with cancer vaccine; **(B)** The tumor growth curve; **(C)** Representative flow cytometric gate of CD45^+^ cells, CD4^+^ T cells, CD8^+^ T cells and CD8^+^IFNγ^+^ T cells; Proportion of CD45^+^ cells **(D)**, CD4^+^ T cells **(E)**, CD8^+^ T cells **(F)** and CD8^+^IFNγ^+^ T cells **(G)** in tumor infiltrating lymphocytes. The threshold for statistical significance was set at p < 0.05 (* p < 0.05, ** p < 0.01, *** p < 0.001, **** P < 0.0001, ns, not significant).

The experiment design is shown in **Figure 3A**. Oral supplementation of LGG alone (VLGG) results in a 38.4% decrease compared to the control group (CTR) in tumor volume, thus hindering tumor growth, which is slightly better than the JP-treated group (VJP group, with a 27.9% reduction in tumor volume compared to CTR). Notably, the group of VLGG-JP (Vaccine+ LGG-JP) exhibits the strongest ability to inhibit tumor growth (**Figure 3B**). The vaccine group (V) starts to fail to the control the tumor on day 28 after tumor induction, with a 13.4% reduction in tumor volume compared to CTR, while the combination of LGG-JP has significantly inhibited tumor growth at day 28, with a 50.6% reduction in tumor volume. The aforementioned findings indicate that the synbiotic of JP and LGG is more effective in inhibiting tumor growth.

The fluorescence-activated cell sorting (FACS) gating strategy of immune cells in tumor microenvironment are shown in **Figure 3C**. There is no obvious change in the percentage of CD45^+^ cells (**Figure 3D**), whereas jujube powder (JP) and *Lactobacillus rhamnosus* GG (LGG) both improve the ration of CD4^+^ T cells (**Figure 3E**) and CD8^+^ T cells (**Figure 3F**) compared with the vaccine alone. Notably, LGG-JP significantly increases the proportion of CD8^+^IFNγ^+^ T cells (**Figure 3G**), which is consistent with the tumor growth curve.

### 3.3 The synbiotic LGG- JP reshaped mice gut microbiota

**Figure 4:**
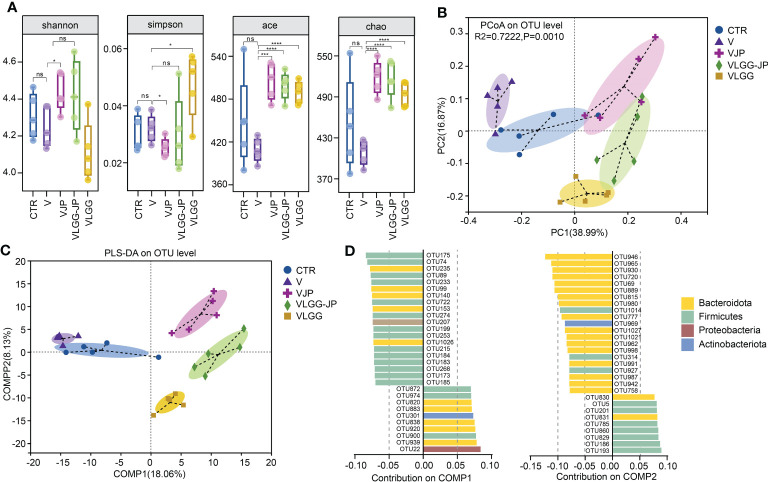
The structure of restored gut microbiota after oral administration with LGG-JP. **(A)** Alpha diversity shown as shannon, simpson, ace and chao index; **(B)** Principal co-ordinates analysis (PCoA) on OTU level; **(C)** Partial Least-Squares Discriminant Analysis (PLS-DA) on OTU level; **(D)** Contribution of microbiota at phylum on COMP1 and COMP2. The threshold for statistical significance was set at p < 0.05 (* p < 0.05, *** p < 0.001, **** p < 0.0001, ns, not significant).

On Day 28, fecal samples were collected from the mice at the end of the experiment for 16S rRNA gene sequence and untargeted metabolomics. As **Figure 4A** shows, there are no significant differences in alpha diversity between the control (CTR) and vaccine group (V). Jujube powder (JP) both significantly increases microbial diversity and richness, as shown by increased Shannon index, ACE index, Chao index and decreased Simpson index. In contrast, although LGG improves community richness, as evidenced by increased ACE and Chao indices, LGG reduces community diversity, as shown by a decrease in the Shannon index and an increase in the Simpson index (**Figure 4A**). It is worth of noting that the synbiotic LGG-JP restores LGG-decreased microbial diversity, Shannon and Simpson index in particular. Principal co-ordinates analysis (PCoA) shows a total of 5 clusters (**Figure 4B**), indicating that both vaccine and oral treatment with either JP or LGG altered the composition of the intestinal microbe. Partial least-squares discriminant analysis (PLS-DA) shows the difference of microbial composition among different groups (**Figure 4C**). Consistently, the microbial composition of the VLGG-JP group was between VJP and VLGG groups. The species contribution on COMP1 and COMP2 shows that the variation between *Bacteroidota* and *Firmicutes* is the main factor (**Figure 4D**).

**Figure 5:**
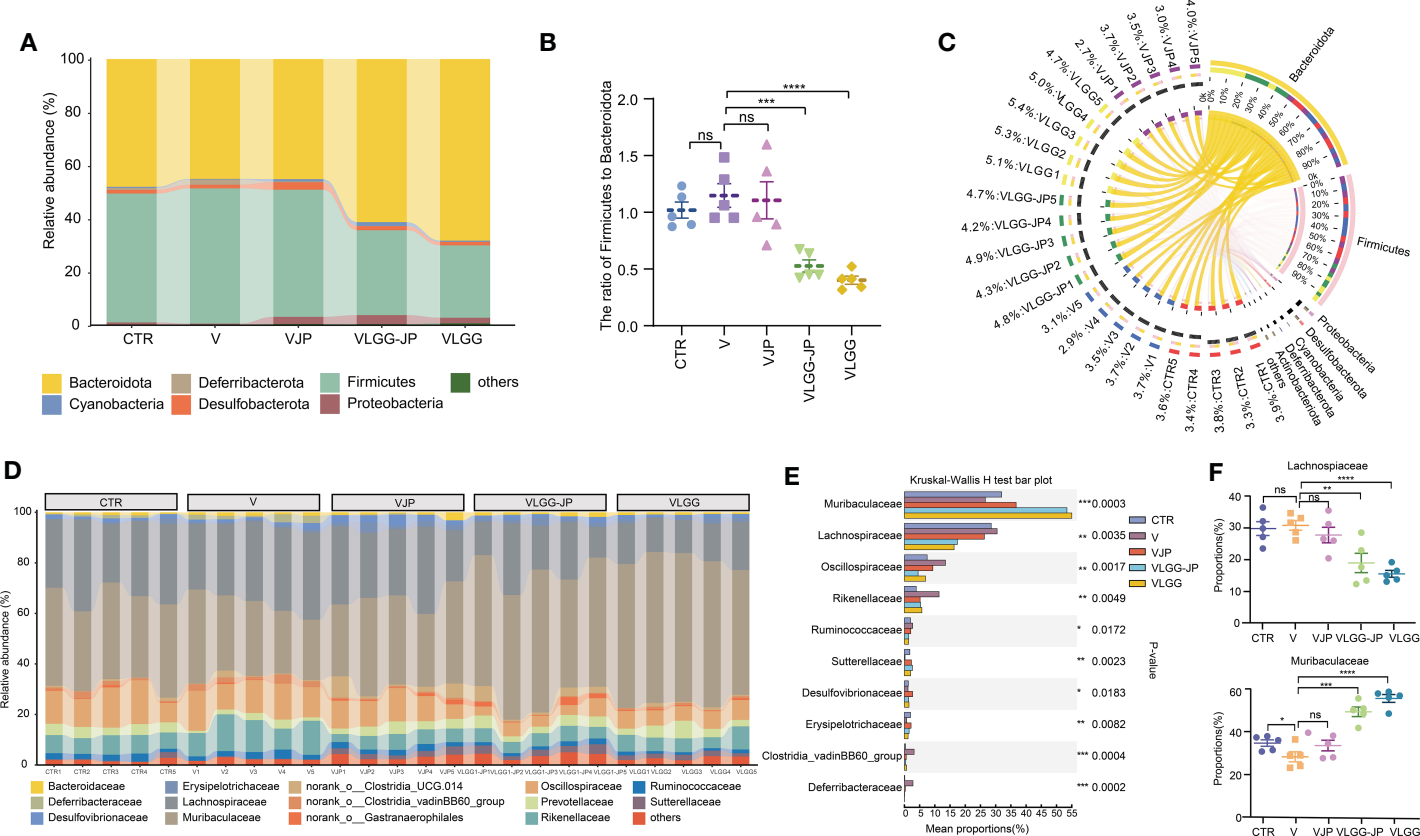
The composition of microbial communities. **(A)** Taxonomic composition of microbial community at phylum; **(B)** The ratio of *Firmicutes* to *Bacteroidota* (F/B); **(C)** Distribution of microorganisms for each sample at phylum level; **(D)** Taxonomic composition of microbial community at family; **(E)** Kruskal-Wallis H test bar plot (The statistically significant differences were calculated using one-way ANOVA, The threshold for statistical significance was set at p < 0.05 (* p < 0.05, ** p < 0.01, *** p < 0.001, **** P < 0.0001, ns: not significant); **(F)** The abundance of *Lachnospiaceae* (Upside) and *Muribaculaceae* (Underside).

We further analyzed the changes in the taxonomic composition of microbial community at phylum and family of each group. At phylum level, LGG extremely upregulates the abundance of *Bacteroidota* while downregulates *Firmicutes* (**Figure 5A**), thus resulting in a lower ratio of *Firmicutes* to *Bacteroidota* (F/B) (**Figure 5B**). The synbiotic LGG-JP weakens this trend. Circos graph exhibits the distribution of microorganisms for each sample at phylum level (**Figure 5C**). The width of the bars representing each phylum illustrates the proportionate abundance of that phylum in the sample, which also suggests that *Bacteroidota* dominates the variation. All these results suggest that the synbiotic LGG-JP reshaped the microbial structure which is different to that obtained by individual treatment with JP and LGG, respectively. **Figure 5D** shows the composition of microorganisms in different groups changes notably at family level, where the *Muribaculaceae* and *Lachnospiraceae* appeared distinct differences (**Figure 5E**). The LGG-JP treatment (VLGG-JP) prominently increases the abundance of *Muribaculaceae* while decreases the abundance of *Lachnospiraceae* compared to the vaccine group (V) (**Figure 5F**). Due to the dominate role of *Muribaculaceae* in *Bacteroidota*, the increase of *Muribaculaceae* resulted in an elevation of *Bacteroidota*, which plays a significant role in energy metabolism and lipid metabolism in the intestine. The treatment of LGG might facilitate the colonization of *Muribaculaceae*, which further enhances vaccine-induced anti-tumor immune response.

### 3.4 The reshaped microbiota with synbiotic of LGG-JP enhanced microbial lipid metabolism

**Figure 6:**
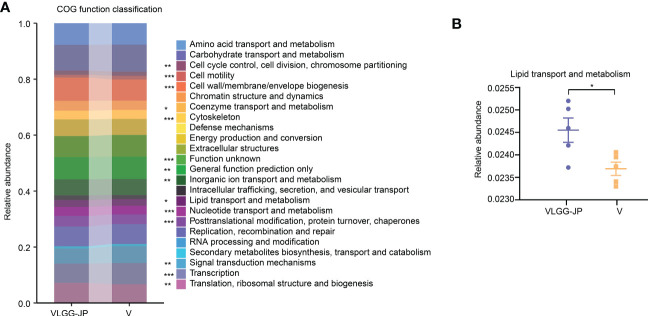
16S function prediction. **(A)** COG function classification on the basis of 16S rRNA gene sequence; **(B)** The relative abundance of lipid transport and metabolism. The threshold for statistical significance was set at p < 0.05 (* p < 0.05, ** p < 0.01, *** p < 0.001).

PICRUSt analysis based on 16S rRNA sequencing data was performed between the vaccine group (V) and the synbiotic treated group (VLGG-JP). As depicted in **Figure 6A**, there are many functional COG modules appear significant differences between these two groups, including chromosome partitioning, cell cycle control and division, cell wall/membrane/envelope biogenesis, etc. Noteworthy, lipid transport and metabolism are substantially elevated in the VLGG-JP group compared with the V group (**Figure 6B**). This may reveal potential pathways through which LGG-JP regulates the gut microbiota and further improves the anti-tumor immune response.

**Figure 7:**
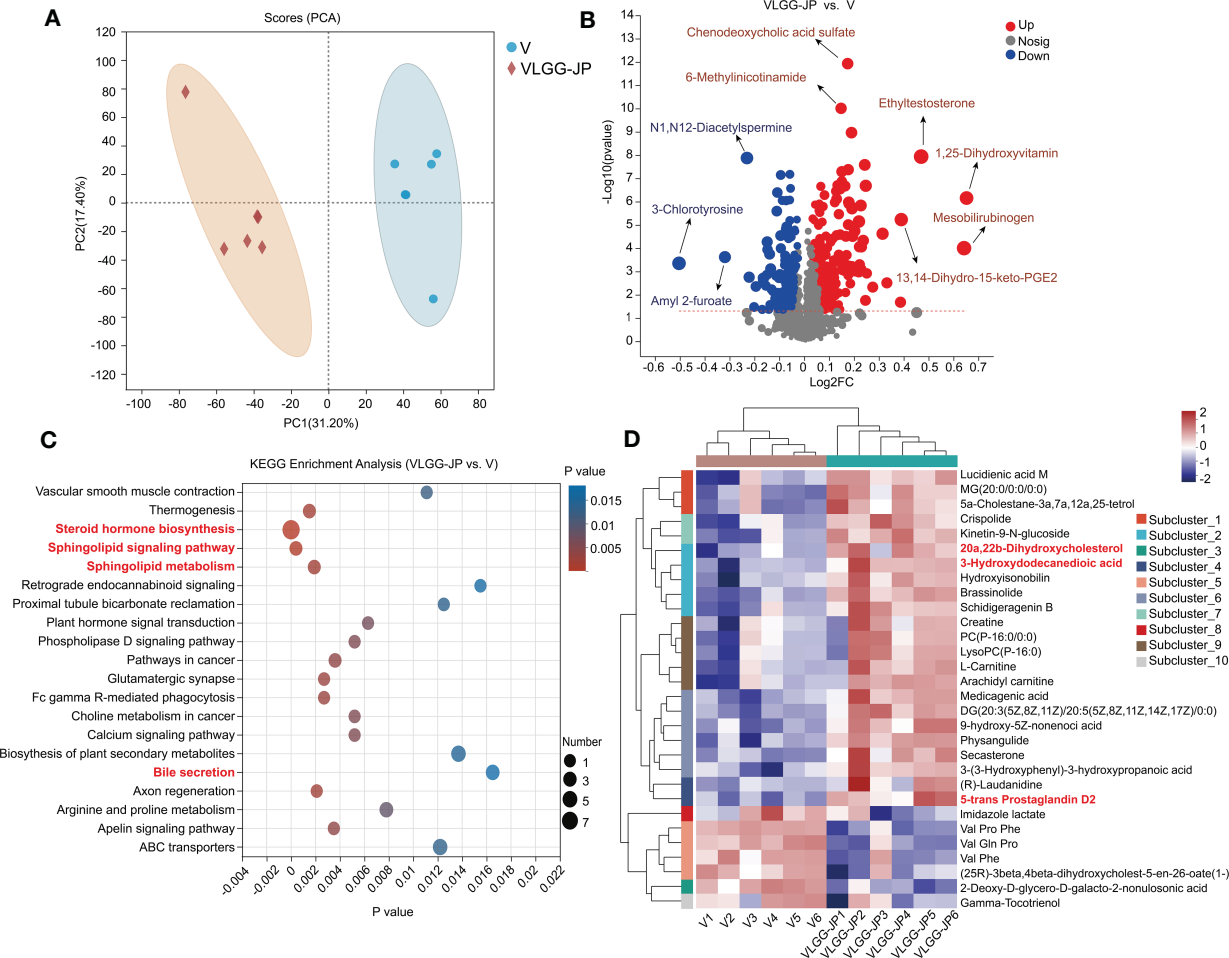
Untargeted metabolomics of mice feces between VLGG-JP group and V group. **(A)** Principal component analysis (PCA) of mice fecal component. **(B)** Differential metabolites volcano gram (Both positive ions and negative ions). The x-axis (Log2FC) represents the fold change in metabolite levels between the two groups, while the y-axis (-Log10(pvalue)) indicates the significance of the difference in metabolite expression. A higher value on the y-axis denotes a more significant expression difference. Each point on the graph corresponds to a specific metabolite, with the size of the point indicating the VIP value (Value Importance in Projection). The points on the left are down-regulated metabolite, while the points on the right are up-regulated metabolites. **(C)** KEGG enrichment analysis. **(D)** Hierarchical clustering analysis of different metabolites.

To further elucidate the mechanism by which gut microbiota affects anti-tumor immunity, we performed untargeted metabolomics of mice feces in the LGG-JP treated group (VLGG-JP) and the vaccine group (V) at the end of the experiment (Day 28). Principal component analysis (PCA) reveals notable variations in metabolites between the VLGG-JP group and the V group, with two distinct clusters (**Figure 7A**). The volcano map shows the distribution of differential metabolites between the two groups (**Figure 7B**). Compared to the vaccine group (V), 74 metabolites upregulate while 85 metabolites downregulate in positive ion mode in the VLGG-JP group. As for negative ion mode, there are 152 metabolites are upregulated and 38 metabolites are downregulated (**Figure 7B**). The metabolites related to bile acid metabolism are significantly upregulated, such as chenodeoxycholic sulfate and mesobilirubinoge, suggesting that bile acid metabolism may have a significant contribution. The biologic pathways enrichment based on KEGG metabolic pathway were analyzed on the online Majorbio Cloud Platform (www.majorbio.com). KEGG enrichment analysis suggests significant changes in lipid metabolism and bile acid metabolic pathways, including: Steroid hormone biosynthesis, Sphingolipid signalling pathway and Sphingolipid metabolism (**Figure 7C**). Meanwhile, ten distinct subclusters are observed among a heatmap based on hierarchical clustering analysis of the top 30 metabolites in abundance (**Figure 7D**), supporting the distinct metabolic feathers between these two groups. Among them, lipid metabolites, i.e., 20a, 20b-Dihydroxycholesterol, 3-Hydroxydodecanedioic acid and 5-trans Prostaglandin D2, have significant differences, which is consistent with the PICRUSt prediction based on the 16S rRNA gene sequences.

## 4 Discussion

The therapeutic cancer vaccine is expected to inhibit tumor growth by stimulating anti-tumor immune response in patients with specific tumor antigens (27). Unfortunately, most therapeutic cancer vaccines failed in phase III clinical trials, with less than 1% success rate. While the effects of gut microbiota on the efficacy of cancer immunotherapy, especially the immune checkpoint blockade (ICB) (10–12) and adoptive immune cell transfer (ATC) (13, 14) are well documented, the effect of gut microbiota on cancer vaccine is poorly understood.

In this work, we used a MC38 mice colon cancer model and employed whole cells cancer vaccine to investigate the impact of gut microbiota on cancer vaccine. We show that the anti-tumor effect of cancer vaccine was impaired in microbiota-depleted mice (**Figure 2B, C**). Recently, Guido and co-workers reported that gut microbiota influenced the anti-tumor activity of neo-epitope-based cancer vaccines in CT26 bearing mice (28). Besides, the whole cells cancer vaccine shows excellent preventive but faint therapeutic effects (**Figure 2B, F**), which is consistent with clinical trials. We proposed a synbiotic composing a natural nutrient (jujube powder, JP) and a probiotic (*Lactobacillus rhamnosus* GG, LGG), and examined its effectiveness in enhancing cancer vaccine treatment. Oral administration of LGG-JP significantly enhanced the effect of cancer vaccine in inhibiting tumor growth (**Figure 3B**), as well as an enhanced the proportion of CD4^+^ T cells and CD8^+^ T cells infiltration in tumor microenvironment (**Figure 3E, F**). The optimal activation of dendritic cells (DCs) by tumor antigens is critical for the efficacy of therapeutic cancer vaccine, which further results in intense and durable responses of CD4^+^ T lymphocytes and cytotoxic CD8^+^ T lymphocytes, as well as the enhanced infiltration in the tumor microenvironment (TME) (29). The study by Wei and co-workers also demonstrated that the anti-tumor activity of anti-PD-1 immunotherapy was enhanced by oral treatment of live LGG through boosting tumor-infiltrating DCs and CD8^+^ T cells (30). Studies listed in **Table 1** confirm that the probiotic strain *Lactobacillus rhamnosus* GG (LGG) may activate CD8^+^ T cells and inhibit the growth of colon cancer cells. Our results show that oral administration of LGG could enhance the anti-tumor activity of cancer vaccines by amplifying CD8^+^ T cells infiltration in tumor microenvironment. Moreover, this effect is further enhanced when LGG is jointed used with jujube powder, forming a synbiotic. Our previous studies have shown that jujube could increase intestinal short chain fatty acid synthesis and enhance tumor infiltration of CD8^+^ T cells, thereby improving the anti-tumor effect of anti-PD-L1 immunotherapy and in MC38 bearing mice (20, 22). Combinations of probiotics and natural products have been found to be beneficial to the immune system, as also reported elsewhere (**Table 1**). Here we propose a new synbiotic composing LGG and jujube powder that can enhance the anti-tumor effect of cancer vaccines.

**Table 1:** Oral administration of LGG is beneficial for immune system.

Intervention method	Intervention results and molecular mechanisms	Reference
LGG	Inhibited the tumor growth in dimethyl hydrazine (DMH)-induced colon cancer in rats	Goldin et al. (31)
LGG	Induced apoptosis of cancer cells and decreased expression of angiogenic proteins in DMH-induced colon cancer	Yaser et al. (32)
LGG	Regulated IL-10 signaling in murine colon by upregulating the IL-10R2 receptor subunit	Mirpuri et al. (33)
LGG	Decreased tumor burden in mouse colorectal cancer by activating colonic CD8^+^ T cells through a Toll-like receptor 2 (TLR2)	Joshua et al. (24)
LGG and soluble corn fiber	Boosted innate immunity in older women by augmenting the activity of natural killer (NK) cells	Costabile et al. (34).
LGG and high-fiber diet	Downregulated pro-carcinogenic and change metabolic that affect the proliferation of cancer cells *in vitro* and *in silico*	Greenhalgh et al. (18)
*Lactobacillus gasseri* 505 and Cudrania tricuspidata leaf	Alleviated colitis-associated colorectal cancer	Nam et al. (17)

Nutritional interventions may influence immune response by altering gut microbiota. Growing evidence suggests that the gut microbiome contributes to the response to cancer immunotherapy. Studies have indicated that alterations in the gut microbiome, such as increased α-diversity, are associated with improved response to immunotherapy in some patients. Although LGG improved anti-tumor activity, it reduced microbial α-diversity (**Figure 4A**). In contrast, jujube powder (JP) could increase microbial α-diversity, thus the combination of JP and LGG restored microbial α-diversity damaged by LGG. The LGG provided live beneficial bacteria, while the JP serve as a food source for other beneficial bacteria, allowing them to thrive and colonize in the gut, which could partially explain why this combination is more effective than using each of them individually. This effect of synbiotic has also been confirmed in clinical trials (35).

There have also been significant changes in species composition after treatment with LGG. The abundance of *Bacteroidota* significantly increased while *Firmicutes* decreased in LGG treated group (**Figure 5A**). Clinical trials have also shown a notable decrease in *Firmicutes* after one month of LGG administration in volunteers (36). The increase in the family of *Muribaculaceae* in the LGG and LGG-JP treatment groups also raised the proportion of *Bacteroidota* (**Figure 5D**). Some members of *Muribaculaceae* are known for their ability to utilize mucus-derived monosaccharides. Commensals that compete with pathogens for nutrients in the gut play an important role as ecological gatekeepers, and are promising candidates for therapeutic interventions (37).

Both 16S functional prediction and metabolomics data show significant changes in lipid metabolism and bile metabolism after oral supplement with LGG-JP (**Figures 6**, **7**). The deviation of lipid metabolism is recognized as a defining characteristic of colorectal cancer (CRC) (38). The accumulation of lipids could stimulate CRC development through elevated levels of reactive oxygen species and MAPK (Mitogen-Activated Protein Kinase) signalling (39). MAPK families occupy a crucial position in orchestrating a range of intricate cellular processes including proliferation, apoptosis, differentiation and transformation, hence the dysregulation of MAPK signalling has been implicated in many diseases such as cancers (40). Of which, bile acids (41), fatty acids (42) and cholesterol (43) have implicated in the development of colorectal cancer (CRC). Recently, Jae Won and colleagues revealed that some bacteria could generate the bile acids and catalyze the final reaction by two special enzymes (44). These two enzymes are encoded only in *Firmicutes* and has been confirmed to increase in CRC patients (45). Our results show a remarkable decrease in the abundance of *Firmicutes* after treatment with LGG or LGG-JP (**Figure 5A**), which might be favourable for cancer treatment. Up to now, little is known about the effect of lipid metabolism and bile acids metabolism on cancer vaccine response. Only Pulendran and colleagues have demonstrated that antibiotic treatment significantly decreased serum secondary bile acids by up to 1,000-fold in vaccinated persons, thereby compromising the efficacy of vaccine-induced specific immune responses (46). On the other hand, it has been established the immune response within the tumor microenvironment (TME) is suppressed, which is closely related to abnormal lipid metabolism. This exerts a substantial influence on the percentage and function of immune cells (47). Michael et al. reported that activated T cells, including CD4^+^ T cells and CD8^+^ T cells, require higher rate of fatty acid oxidation to maintain energy supply, hence the level of fatty acids in cells plays a crucial part to maintain the intracellular fatty acid oxidation (48). Our results suggest that oral supplement with LGG-JP improved lipid metabolism in intestinal microorganisms and intensified infiltration of CD8^+^ T cells in TME. The interaction between these two remains to be studied in depth. Future studies can elucidate the molecular mechanisms by immunohistopathology, thereby providing a deeper understanding of the underlying pathways. The global impact of lipid metabolism on cancer vaccine needs further investigation.

## 5 Conclusion

In conclusion, this study highlights the impacts of gut microbiota on the therapeutic outcome of cancer vaccine. Oral administration of a synbiotic composing *Lactobacillus rhamnosus* GG (LGG) and jujube powder (JP) significantly enhanced the therapeutic efficacy of whole tumor cells vaccine in mice bearing MC38 colon cancer cells. Such synbiotic improved the α-diversity of the microbial community and enhanced lipid metabolism which further reinforced lymphocyte infiltration in the tumor microenvironment, especially CD8+ T cells. These findings open up new avenues for exploring the use of dietary interventions, such as synbiotics, to enhance the efficacy of cancer immunotherapy and provide a foundation for future translational studies on the potential of synbiotics as a complementary approach to cancer treatment. The inclusion of interventions in dietary guidelines for cancer prevention and treatment may improve patient outcomes and reduce the burden of cancer. The specific mechanism by which prebiotic intervention in gut microbiota affects the therapeutic effect of cancer vaccine needs to be further clarified.

## Data availability statement

The 16S rRNA sequencing dataset was deposited in the BioProject database of NCBI with accession PRJNA939012. All data that support the findings of this study are available upon reasonable request from the corresponding authors.

## Ethics statement

The animal study was reviewed and approved by Institutional Animal Care and Use Committees of Tsinghua University.

## Author contributions

NJ: Conceptualization, Formal analysis, Writing- Original draft preparation. LW: Conceptualization, Methodology, Writing - Review & Editing. HZ: Investigation. GJ: Conceptualization, Writing - Review & Editing. ZL: Conceptualization, Supervision, Writing - Review & Editing. All authors contributed to the article and approved the submitted version.
