# Supplementary material for: Enhancing therapeutic effects of murine cancer vaccine by reshaping gut microbiota with Lactobacillus rhamnosus GG and jujube powder
Source: Front Immunol. 2023 Jun 26;14:1195075. doi: 10.3389/fimmu.2023.1195075 (PMC10332846; doi:10.3389/fimmu.2023.1195075)
Supplement: Supplementary file 1 [file Table_1.docx]

Supplementary Material

**Supplementary Table 1.** The elution procedure of mobile phase

| Time (min) | Flow rate (mL/min) | A (%) | B (%) |
| --- | --- | --- | --- |
| 0 | 0.4 | 100 | 0 |
| 0.1 | 0.4 | 95 | 5 |
| 2 | 0.4 | 75 | 25 |
| 9 | 0.4 | 0 | 100 |
| 13 | 0.4 | 0 | 100 |
| 13.1 | 0.4 | 100 | 0 |
| 16 | 0.4 | 100 | 0 |

**Supplementary Table 2.** The mass spectrometry parameters

| Description | Parameters |
| --- | --- |
| Scan type (m/z) | 70-1050 |
| Sheath gas flow rate (arb) | 40 |
| Aux gas flow rate (arb) | 10 |
| Heater temp (℃) | 400 |
| Capillary temp (℃) | 320 |
| Spray voltage (+) (V) | 3500 |
| Spray voltage (-) (V) | -2800 |
| S-Lens RF Level | 50 |
| Normalized collision energy (eV) | 20,40,60 |
| Resolution (Full MS) | 70000 |
| Resolution (MS^2^) | 17500 |
